# Acupuncture improves the residual urine volume of bladder of middle age patients with urinary retention post-stroke: A protocol for systematic review and meta analysis

**DOI:** 10.1097/MD.0000000000031697

**Published:** 2022-11-25

**Authors:** Di Cao, Qiguang Yang, Fengjun Qi, Shuhong Gu, Tao Yu, Lin Zhu, YiMing Liu, Junjing Gui, Baoru Yang, Xiaolin Zhang

**Affiliations:** a Department of Rehabilitation, The Second Affiliated Hospital of Changchun University of Chinese Medicine (Changchun Hospital of Chinese Medicine), China; b Hubei University of Chinese Medicine, China; c Acupuncture and Massage Center of The Third Affiliated Hospital of Changchun University of Chinese Medicine, China.

**Keywords:** acupuncture, post-stroke, protocol, residual urine volume, systematic review, urinary retention

## Abstract

There are 15 million new cases of stroke every year in the world, 65% of which have dysuria in the early stage of stroke, which seriously affects the quality of life of stroke patients. The purpose of this study is to evaluate the results of randomized controlled trials to determine whether acupuncture can improve the residual urine volume of the bladder in middle age patients with urinary retention post-stroke.

**Methods:**

Eight databases, including China National Knowledge Infrastructure, Chinese Scientific Journal Database, Cochrane Central Register of Controlled Trials, Embase, MEDLINE, PubMed, Wanfang Database, and Web of Science, will be searched using English and Chinese search strategies. In addition, manual retrieval of research papers, conference papers, ongoing experiments, internal reports, etc, will supplement electronic retrieval. All eligible studies published on or before October 1, 2022 will be selected. To enhance the effectiveness of the study, only clinical randomized controlled trials related to the use of manual acupuncture for the treatment of urinary retention post-stroke will be included.

**Conclusion:**

The residual urine volume of bladder will be the primary outcome measure, whereas the Clinical efficiency will be the secondary outcomes. Side effects and adverse events will be included as safety evaluations. To ensure the quality of the systematic evaluation, study selection, data extraction, and quality assessment will be independently performed by two authors, whereas a third author will resolve any disagreement.

## 1. Introduction

At present, there are 15 million new cases of stroke in the world every year, 65% of which have urinary dysfunction in the early stage of stroke, including frequent urination, urgency, incontinence and urinary retention.^[1]^ The occurrence of urinary retention post-stroke is related to the decrease of detrusor contractility^[2]^ and dyssynergism of detrusor and external sphincter caused by stroke affecting the dominant cerebral hemisphere and spasm of external sphincter of urethra, which seriously affects the quality of life of patients.^[3]^ Currently commonly used in clinical practice α Receptor blockers and cholinergic drugs are used for treatment, but some patients have no obvious effect, and must use indwelling catheter for intermittent catheterization, which increases the risk of infection and complications, and even leads to other bladder dysfunction.^[4]^ From the perspective of Chinese medicine, urinary retention post-stroke belongs to the category of “dysmenorrhea,” which results in poor urination due to the loss of bladder gasification function.

Systematic acupuncture at corresponding acupoints can regulate the function of bladder gasification, thus improving the state of urine retention. Many studies^[5–10]^ have shown that acupuncture can effectively improve the state of urinary retention post-stroke, Some studies^[11,12]^ have reported that acupuncture can shorten the treatment time of urinary retention and reduce the residual urine volume of the bladder. This study has not been summarized.

Therefore, this study intends to use the systematic evaluation method to analyze the clinical randomized controlled study of acupuncture treatment on post-stroke urinary retention, so as to clarify the efficacy of various types of acupuncture intervention methods on post-stroke urinary retention, and explore the impact of acupuncture on bladder residual urine volume, so as to provide a judgment basis for clinical decision-making.

The proposed date for the completion of this study is October 28, 2023.

## 2. Methods

### 2.1. Study registration

This protocol is based on the Preferred Reporting Items for Systematic Reviews and Meta-Analyses Protocols guidelines^[13]^ and the corresponding checklist. The systematic review protocol is registered in PROSPERO with the registration number of CRD42022363395, and the specific plan has been published on its official website (https://www.crd.york.ac.uk/prospero/display_record.php? RecordID = 363395). This study will be carried out in strict accordance with the preliminary design framework.

### 2.2. Inclusion criteria for study selection

#### 2.2.1. Types of studies.

All randomized controlled trials (RCTs), and quasi-randomized controlled trials will be included. Excluded from the meta-analysis are duplicated publications; the control group also used acupuncture intervention, studies with unavailable or in correct data, articles not reporting outcomes of interest. Also excluded are studies enrolling <30 participants.

#### 2.2.2. Types of participants.

Patient inclusion criteria will include: Patients’ age will be between 46 and 69 years old,^[14]^ regardless of their sex and nationality; Conforming to the diagnostic criteria of Western medicine stroke, diagnosed by computed tomography or magnetic resonance imaging, and any one or more of the clinical manifestations of urinary retention; it is consistent with the above diagnostic criteria of stroke and accompanied with clinical manifestations of urinary retention; the course of the disease was >1 month, but ≤6 months; the residual urine volume of bladder was >50 mL by B-ultrasound^[15]^; clear consciousness, stable vital signs, no obvious cognitive and language dysfunction; before stroke, the patients could urinate normally without bladder dysfunction.

#### 2.2.3. Types of interventions.

We will include studies in which the intervention group received acupuncture or acupuncture combined with basic treatment (Neurology treatment or catheterization or bladder rehabilitation training), while the control group was treated with sham acupuncture, placebo acupuncture, drugs or basic treatment.

#### 2.2.4. Types of outcome measures.

The primary outcome measure will be the residual urine volume of bladder measured by B ultrasound. The secondary outcome measures will include the cure rate (bladder residual urine <50 mL) or effective rate (bladder residual urine volume <100 mL), adverse drug reaction, and adverse events.

Ischemic stroke and hemorrhagic stroke will be analyzed in subgroup analysis.

### 2.3. Search methods for the identification of studies

#### 2.3.1. Data sources.

A structured and systemic literature search for eligible and relevant articles published on or before October 1, 2022 will be conducted. The following databases will be searched: China National Knowledge Infrastructure, Chinese Scientific Journal Database, Wanfang Database, Cochrane Central Register of Controlled Trials, Embase, MEDLINE, PubMed, and Web of Science. The language of the selected literature will include Chinese and English. The search terms include cerebral apoplexy, sequelae of stroke, post-stroke, urinary retention, residual urine volume of bladder, acupuncture, clinical RCT.

#### 2.3.2. Searching other resources.

**Figure 1.**
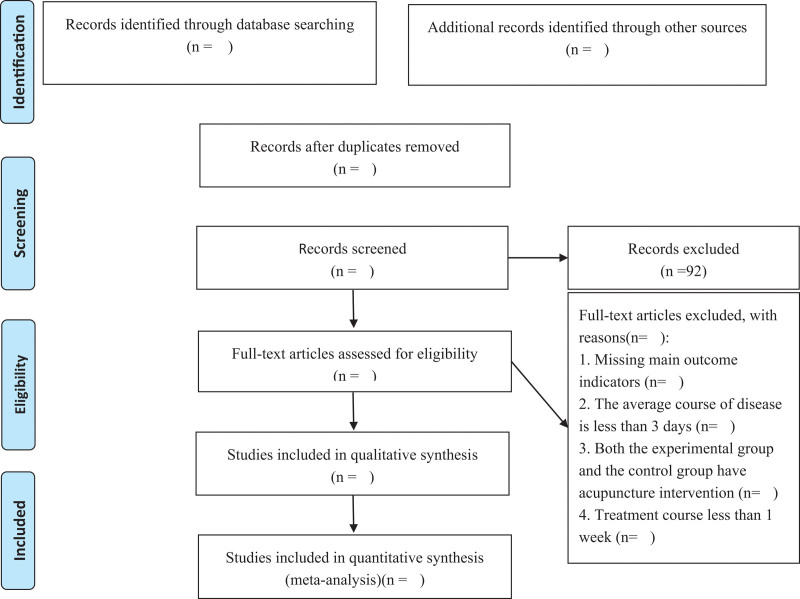
Preferred Reporting Items for Systematic Reviews and Meta-Analyses (PRISMA) flow diagram of study and exclusion.

The manual search will mainly be used in searching for relevant studies. Details of the selection process are shown in the flow chart and the screening process is summarized in a flow diagram (Fig. 1).

#### 2.3.3. Search strategy.

The search strategy will be based on the Cochrane handbook guidelines (5.1.0) and will include keywords, such as “post-stroke” or “after stroke,” “acupuncture” or “manual acupuncture,” “urinary retention” or “residual urine volume of bladder,” and “clinical RCT.” Subsequent searches will involve the use of MeSH headings, including “post-stroke,” “urinary retention,” and “manual acupuncture,” in addition to keywords from the initial retrieval. Additional article searches will involve the review of the reference lists of relevant research articles. As an example, the search strategy for PubMed is summarized in Table 1.

**Table 1:** Search strategy for PubMed.

Number	Search terms
#1	“Urinary retention” [Mesh]
#2	Urinary retentions [Title/Abstract]
#3	or #1–#2
#4	Post-stroke [Title/Abstract]
#5	After-stroke [Title/Abstract]
#6	or #4–#5
#7	Residual urine volume of bladder [Title/Abstract]
#8	“Acupuncture” [Mesh]
#9	Acupuncture [Title/Abstract]
#10	or #8–#9
#11	Randomized controlled trial [Publication type]
#12	Controlled clinical tria [Publication type]
#13	or #11–#12
#14	#3 and #6 and #7 and #10 and #13

### 2.4. Data collection and analysis

#### 2.4.1. Selection of studies.

Two researchers (YML and BRY) will independently select the eligible literature according to the inclusion and exclusion criteria after reading their titles and abstracts. Subsequently, the full texts of the papers will be read and uncontrolled research, nonrandomized studies, and studies with inconsistent evaluation criteria or similar data will be excluded. If any differences occur during the screening, the third author (DC) would intervene.

#### 2.4.2. Data extraction and management.

Two researchers (JJG and ZL) will use a predesigned data extraction table to extract the data of the included studies. The extracted data will include author, year, sample size, course of treatment, intervention measures, outcome indicators, adverse reactions, etc. The study selection procedure will be performed according to the Preferred Reporting Items for Systematic Reviews and Meta-Analyses guidelines, which are presented in the flow diagram.

#### 2.4.3. Statistical analysis.

We will use Statistics Analysis System and Stata to analyze the Standard Deviation, Standard Error and Mean of each group. When we encounter the literature with missing data, we will try to contact the author, if we can’t get the full data through contact. Then we will exclude the author’s full data. We will use the Review Manager 5.3 software provided by Cochrane collaborative network for statistical analysis. For continuous variables, the mean and standard of each study will be obtained and pooled as a mean difference or a standardized mean difference with a 95% confidence interval. The statistical heterogeneity of the included clinical RCTs will be analyzed. Cochrane *I*^2^ test will be used to test for heterogeneity. When *I*^2^ is <50% or *P* > .05, it indicates that there is no statistical heterogeneity between studies.^[16]^ The fixed-effects model will be selected to combine the effect amount; otherwise, the random effects model will be adopted.

#### 2.4.4. Methodological assessment of quality.

The qualities of the included studies will be evaluated using the risk of bias table proposed by Cochrane collaborative network. The risk table includes 6 items: random sequence generation mode, whether to use allocation concealment, whether to blind the subjects and intervention providers, whether to blind the result evaluators, whether the result data are complete, whether to select the result report, and other bias sources. The criteria used to assess the risk of bias are “low risk,” “high risk,” and “unclear.”^[16]^ Two evaluators will independently evaluate the methodological qualities of the studies. In cases of disagreement, the third author would intervene.

#### 2.4.5. Assessment of heterogeneity.

If there is no significant heterogeneity (*I*^2^ < 50%) between a group of studies, the fixed-effects model will be used for evaluation. If there is significant heterogeneity (*I*^2^ > 50%) between a group of studies, the random-effects model will be used for evaluation.^[16]^ Sensitivity analysis or subgroup analysis will then be conducted as required to explain the heterogeneity.

#### 2.4.6. Subgroup analysis.

If possible, subgroups will analyzed according to Brain hemorrhagic type and cerebral ischemic type or different acupuncture manipulation.

#### 2.4.7. Sensitivity analysis.

When possible, we will perform sensitivity analysis to explore the effects of a trial’s risk of bias on primary outcomes. Lower quality trials will be excluded from these analyses and the meta-analyses will be repeated according to sample size and insufficient data to assess quality and robustness when significant statistical heterogeneity arises.

#### 2.4.8. Assessment of publication bias.

If >10 trials meet the study criteria, we can use RevMan 5.3 software to draw and analyze the funnel chart and use the funnel chart to evaluate the potential publication bias.

#### 2.4.9. Grading the quality of evidence.

The Grading of Recommendations Assessment, Development and Evaluation approach^[17]^ is recommended for the analysis of the level of evidence.

## 3. Discussion

Research^[18]^ shows that the probability of uroschesis post-stroke is as high as 79%. Nakayama et al^[19]^ also pointed out that about 50% of stroke patients will suffer from urinary retention. Patients may have less abdominal distension, frequent urination, painful urination, poor urination, or even blocked urination. At present, urethral catheterization is often used clinically. Although it can quickly relieve symptoms, it is easy to damage the urethra, induce urinary system infection, prolong the bedtime, increase the pain of patients, and hinder the rehabilitation training of patients with limb paralysis post-stroke, which seriously affects the quality of life of patients.^[20]^

According to Chinese medicine theory, urinary retention post-stroke is a kind of urosis caused by stroke, which is mainly related to the loss of brain spirit post-stroke, the inability of spirit to channel qi, and the disadvantageous gasification of kidney and bladder. Chinese medicine has unique advantages in treatment. Acupuncture and moxibustion treatment has the advantages of simple operation, wide range of adaptation, significant curative effect, high safety coefficient and small adverse reactions. “The Expert Consensus on Diagnosis and Treatment of Neurogenic Bladder post-stroke” points out that acupuncture treatment has the advantages of less pain for patients, easy operation for doctors and economy.^[21]^

The study found that acupuncture can affect the activities of peripheral afferent or efferent nerves that innervate the bladder and urethra, so as to regulate the sacral medullary micturition center and supraspinal center, and regulate the bladder function to promote the micturition function of patients with urinary retention post-stroke.^[22]^

At the same time, relevant clinical studies^[23]^ have proved that acupuncture treatment can increase the number of T lymphocyte subsets, reduce the hospital infection rate post-stroke, and enhance the immune function of patients. We have also observed in clinical practice that acupuncture can significantly improve local blood circulation, effectively promote the recovery of the central and surrounding two damaged nerves, if the needle sensation is transmitted to the vulva and directly excites qi to the pudendal meridian and the urethral sphincter it controls, so as to adjust bladder dysfunction, which has reached the therapeutic purpose, and relieve pain for our patients.

It can be seen that acupuncture has a good clinical effect on improving the residual urine volume of bladder in patients with urinary retention post-stroke, and it is easy to operate, which can better improve the quality of life of patients. However, the evidence of evidence-based medicine on acupuncture and moxibustion for the treatment of bladder residual urine volume in patients with urinary retention post-stroke is not sufficient, and the clinical guidance role needs to be improved. Therefore, this study will comprehensively analyze the effect of acupuncture and moxibustion on the improvement of bladder residual urine volume in patients with urinary retention post-stroke, and analyze the deficiencies and prospects of related research. The results of this study can provide scientific basis for acupuncture and moxibustion to improve the residual urine volume of bladder in patients with urinary retention post-stroke, and is conducive to the optimization of clinical acupuncture and moxibustion to improve the diagnosis and treatment scheme of urinary retention post-stroke. It has positive significance for the promotion of acupuncture therapy and the exploration of new management strategies for urinary retention post-stroke.

However, during the progress of the study, bias and significant heterogeneity may occur due to the quality of evidence, efficacy evaluation criteria, reports of adverse reactions, and the absence of long-term follow-up, which may affect the reliability of the study results.

## 4. Limitations

The measurement of residual urine volume by B-mode ultrasound is an important part of evaluating voiding dysfunction. Accurate determination is important for diagnosis and treatment of urinary retention. The evidence of bladder residual urine volume in this study is limited. It is expected that most studies choose to use composite indicators to divide the curative effect. There may be few studies using specific values to record bladder residual urine volume, which will affect the final results.

## 5. Statement

It is not necessary for ethical approval because this article is based on previously conducted studies and does not involve any new studies of human or animal subjects performed by any of the authors. The protocol will be disseminated in a peer-reviewed journal or presented at a relevant conference. All relevant data are within the article and its supporting information files.

## Author contributions

**Conceptualization:** Di Cao, Qiguang Yang, Fengjun Qi, Xiaolin Zhang.

**Data curation:** Lin Zhu, YiMing Liu, Junjing Gui, Baoru Yang.

**Methodology:** Qiguang Yang, Shuhong Gu, Tao Yu.

**Writing – original draft:** Di Cao, Fengjun Qi, Xiaolin Zhang.

**Writing – review & editing:** Xiaolin Zhang.
